# Alpha-Lipoic Acid Protects Co-Exposure to Lead and Zinc Oxide Nanoparticles Induced Neuro, Immuno and Male Reproductive Toxicity in Rats

**DOI:** 10.3389/fphar.2021.626238

**Published:** 2021-07-08

**Authors:** Monika S. Deore, Keerthana S, Saba Naqvi, Anoop Kumar, S. J. S. Flora

**Affiliations:** Department of Pharmacology and Toxicology, National Institute of Pharmaceutical Education and Research (NIPER-R), Raebareli, India

**Keywords:** α-lipoic acid (ALA), lead (Pb), zinc oxide nanoparticles (ZnO NPs), oxidative stress, immunoglobulins, reproductive toxicity

## Abstract

We evaluated the neuro-, immuno-, and male reproductive toxicity of zinc oxide nanoparticles (ZnO NPs) alone and in combination with lead acetate. We also studied the therapeutic role of α-lipoic acid postexposure. Lead (10 mg/kg, body weight), ZnO NPs (100 mg/kg, bwt) alone, and their combination were administered orally in Wistar rats for 28 days, followed by the administration of α-lipoic acid (15 mg/kg, bwt) for the next 15 days. Our results demonstrated protective effects of α-lipoic acid on lead and ZnO NP–induced biochemical alterations in neurological, immunological, and male reproductive organs in rats. The altered levels of blood δ-aminolevulinic acid dehydratase (ALAD), immunoglobulins (IgA, IgG, IgM, and IgE), interleukins (IL-1β, IL-4, and IL-6), caspase-3, and tumor necrosis factor (TNF-α) were attenuated by lipoic acid treatment. Lead and ZnO NP–induced oxidative stress was decreased by lipoic acid treatment, while a moderate recovery in the normal histoarchitecture of the brain section (cortex and hippocampus) and testes further confirmed the neuro- and male reproductive toxicity of lead and ZnO NPs. We also observed a significant decrease in the blood metal content in the animals treated with lipoic acid compared to the lead-administered group, indicating the moderate chelating property of lipoic acid. It may thus be concluded that lipoic acid might be a promising protective agent against lead and ZnO NP–induced alterations in the neurological, immunological, and reproductive parameters.

## Introduction

Lead is a widespread highly toxic heavy metal. It is ubiquitous in the environment and generated in various human activities such as mining, burning fossil fuels, paints, printing, gasoline, water pipes, car batteries, cosmetic products, shielding for X-ray machines, and toys. (Mahmood et al., 2012). Its continuous use in various products leads to its accumulation in the environment and serious health hazards as it does not degrade into the environment and remains absorbed into the soil. Lead has neurotoxic and immunotoxic potential in addition to its effects on other organ systems depending upon the dose, route, duration of exposure, etc.

Zinc oxide nanoparticles (ZnO NPs), on the other hand, are widely used in biological applications over other metal oxide nanoparticles due to their excellent biocompatibility, cheapness, and low toxicity (Zhang et al., 2013). Various reports have shown the anticancer, antibacterial, antidiabetic, and anti-inflammatory activities of zinc oxide nanoparticles. These particles are widely used in sunscreens, cosmetics, and bottle coating due to their ultraviolet blocking features (Klaine et al., 2008). However, the toxicity of these particles has also been reported as they can penetrate the individual cells and their nuclei (Wang et al., 2008). The toxic effects of these particles depend upon the size, dosage, duration of exposure, etc. (Keerthana and Kumar, 2020).

Chelation is the most effective strategy currently available to manage the toxicity of metals; however, some important issues need to be raised, such as high therapeutic costs, toxicity, and patient’s quality of life. Thus, there is a need for alternative strategies against metal-induced toxicity (Flora et al., 2008).

Alpha-lipoic acid (ALA), also known as thioctic acid and 1,2-dithiolane-3-pentanoic acid, is a hydrophilic and hydrophobic natural compound widely distributed in cellular membranes and cytoplasm in plants and animals. Its natural occurrence in the human diet is insufficient; therefore, the human body synthesizes it naturally in the liver, heart, and testis to form the required ALA (Jan et al., 2015). It also protects the cellular membranes by interacting with vitamin C and glutathione, which subsequently recycles vitamin E (Laher, 2011). Additionally, it also has a metal-chelating ability besides its role as a potent antioxidant (Kurutas, 2016). It involves the energy metabolism of proteins, carbohydrates, and fats. Physiologically, it disposes of blood glucose and converts energy into ATP (Wesselink et al., 2019). It is a naturally occurring cofactor for the mitochondrial enzymes pyruvate dehydrogenase and α-ketoglutarate dehydrogenase (Kohlmeier, 2003). It has been used as a dietary supplement, multivitamin formula, antiaging supplement, and even as a pet feed. Its therapeutic potential has been investigated and supports its use in diseases like diabetic polyneuropathies, vascular disease, hypertension, inflammation, diabetes, cardiovascular diseases, neurodegenerative diseases, autoimmune diseases, cancer, and AIDS (Shay et al., 2009; Fenga et al., 2017). The literature also suggests the potential use of ALA against metal-induced toxicity. To the best of our knowledge, there is no report available regarding the beneficial effects of ALA against conditions where humans or animals are coexposed to lead and ZnO NPs and the associated neurological, immunological, and male reproductive disorders. In the present study, we thus explored the effect of alpha-lipoic acid in rats exposed to ZnO NPs and lead, alone and in combination.

## Materials and Methods

### Drugs and Chemicals

Zinc oxide nanopowder (∼70 nm particle size), lead (II) acetate trihydrate (99.999% trace metals basis), Freund’s adjuvant (complete), and (±)-α-Α-lipoic acid were purchased from Sigma-Aldrich (St. Louis, Missouri, United States). All ELISA kits were purchased from ELK (Wuhan) Biotechnology Co., Ltd., Hubei, P.R.C. Zinc oxide nanoparticles were purchased from Sigma-Aldrich Chemicals Co., St. Louis (United States).

### Characterization of Zinc Oxide Nanoparticles by Transmission Electron Microscopy (TEM), Dynamic Light Scattering, and Zeta Potential Measurement

The size and morphology of purchased nanoparticles (NPs) were determined using a TEM (Jel JEM 1400, Jeol Ltd., Tokyo, Japan) at the CSIR-Central Drug Research Institute, Lucknow, India. One drop of the ZnO NPs was taken and put onto the carbon-coated copper grid and left for 10 min. The excess amount of sample was removed carefully and then negative staining was done using 2% phosphotungstic acid (PTA). The samples were air-dried for 15 min and viewed under the TEM. The average particle size and size distribution of the ZnO nanoparticles were determined using a Nanosize 90 ZS (Malvern Instruments, United Kingdom) at the National Institute of Pharmaceutical Education and Research (NIPER), Raebareli, Lucknow, India. The measurements were carried out using a disposable cuvette and keeping the temperature at 25°C throughout the experiment. The prepared nanoparticles were diluted with triple distilled water and measured using a Zetasizer.

### Animals

Male Wistar rats (100–120 g) were purchased from the Animal House Facility of the CSIR-Central Drug Research Institute, Lucknow, India. These animals were kept in a quarantine area of our institute’s animal house facility for a week with free access to drinking water and animal feed (standard Chao pellets from ATNT, Germany). The animals were acclimatized for 7 days before their use in experiments, and free access to the standard diet and water continued throughout the study. After the acclimatization, the animals were randomized and housed in an air-conditioned room (room temperature of 25 ± 2°C), 30–70% relative humidity with an alternate 12-h light/dark cycle. All the experimental protocols included in this study were duly approved by the Institutional Animal Ethics Committee (IAEC) of the National Institute of Pharmaceutical Education and Research, Raebareli (Reg. No-IAEC/29/Aug 2019), and the animals were taken care of according to the guidelines provided by the Committee for the Purpose of Control and Supervision of Experiments on Animals (CPCSEA), Ministry of Fisheries, Animal Husbandry, and Dairying, Government of India. All behavioral experiments were performed at the same time daily.

### Experimental Protocol

The animals were divided equally into four groups, and their dosage regimens were as follows:

Group I: normal control;

Group II: ZnO NP (100 mg/kg/b.w., p. o.);

Group III: lead acetate (10 mg/kg/b.w., p. o.);

Group IV: lead acetate and ZnO NP (as described in Group II and III).

**FIGURE 1:**
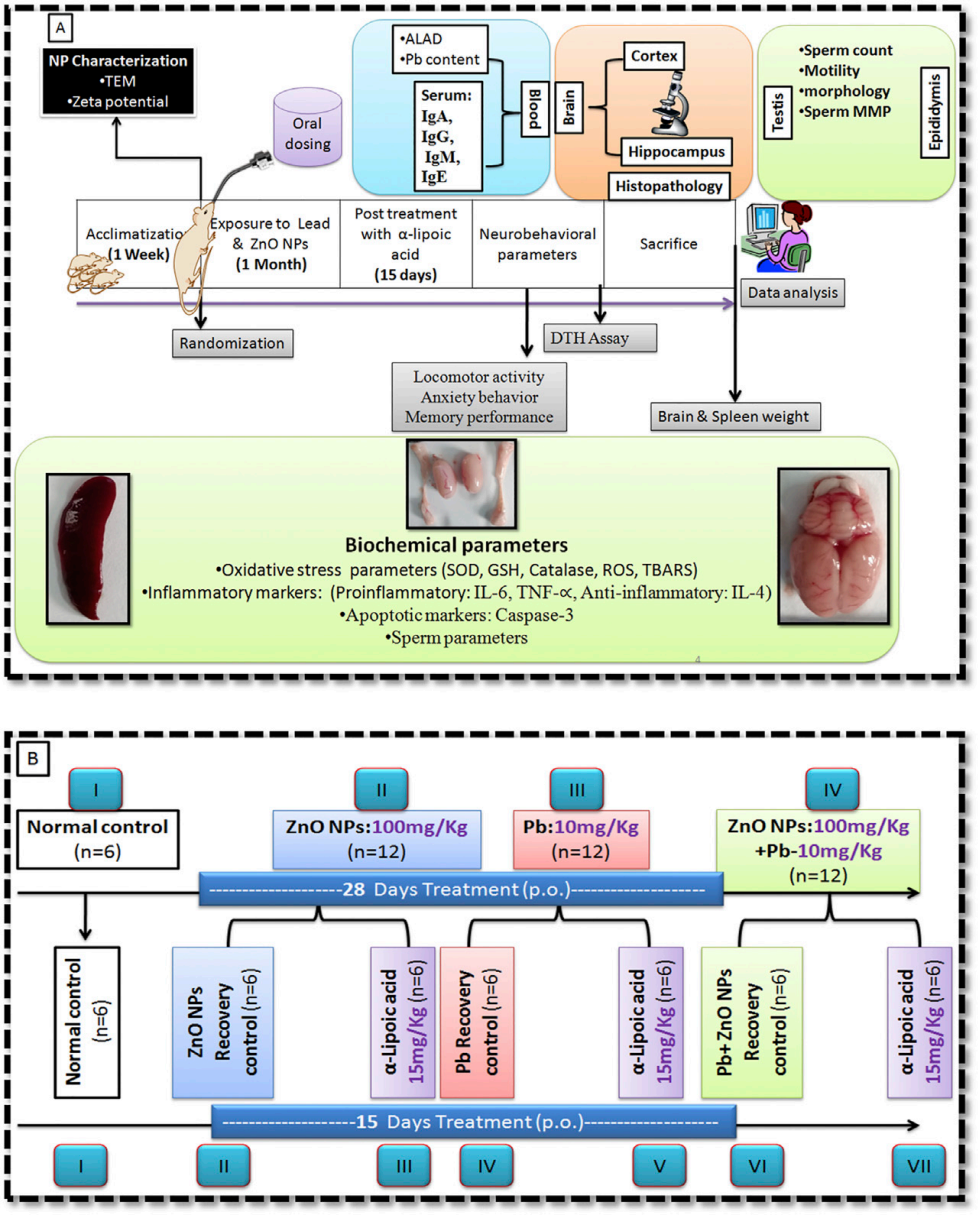
Schematic representation of work plan. **(A)** Experimental design. **(B)** Animal groupings.

After 28 days of exposure, all the animals (except the control group) were further subdivided, each containing six rats (Figure 1B). One group served as an α-lipoic acid treatment group, and the other was a recovery group. Treatment with α-lipoic acid continued for 15 days at a dose of 5 mg/kg/b.w., p. o. The details of the experimental design of the study have been presented in Figure 1. The dosage was selected for the groups as reported in the literature and based on LD_50_ (Gurer et al., 1999). Delayed type hypersensitivity response (Abass et al., 2017) and neurobehavioral parameters were performed after completion of the treatment.

On the last date of sacrifice, animals were deprived of food and fasted overnight. The rats were sacrificed by cervical dislocation, and blood was collected through a retro-orbital puncture, transferred in heparinized tubes, and used for the estimation of serum immunoglobulins (IgA, IgG, IgM, and IgE) and blood metal estimation. The heparinized blood was centrifuged for 15 min at 2500 rpm to collect serum which was used for the estimation of immunoglobulins. The brain, male reproductive organs, and spleen were excised from the body and rinsed in ice-cold saline, and organ weights were recorded according to the calculated body-to-organ ratio after wiping with blotting paper. The brain, spleen, and male reproductive organs were used as target organs for evaluating neurotoxicity, immunotoxicity, and male reproductive toxicity, respectively.

The 10% (w/v) homogenates of the brain, spleen, and testes were prepared, followed by centrifugation at 10,000 *g* for 15 min, and the supernatant was collected and used for the estimation of various biochemical parameters.

### Neurobehavioral Evaluations

#### Assessment of Spontaneous Locomotor Activity

Spontaneous locomotor activity in the treated rats was evaluated using an Optovarimex-4 (Columbus Instruments, Columbus, Ohio, United States) with a slight modification in the method described by Bernal-Morales and colleagues (Bernal-Morales et al., 2017). It consists of a large square-shaped glass chamber with photocells that send continuous unseen infrared light beams horizontally and vertically. The automatic recording was done when the animals interrupted this light beam due to its movement. The experiments were performed for a total of 10 min wherein the resting time and distance traveled by the animal were recorded.

#### Elevated Plus Maze

It consists of a total of four arms (two open and two closed), with walls approximately 15 cm high and an open roof. A camera (HD Logitech C525) was set on the apex of the maze. The rats were carried into the procedure room in the home cages with maintained dark conditions. The animals were kept to the spontaneous exploration of the maze for 5 min by placing them into the center facing an open arm. The software ANY Maze was used to record the retention time and number of open arm entries of the animals. The apparatus was cleaned every time with 70% ethanol to remove any olfactory stimulus (Walf and Frye, 2007).

#### Learning and Memory in the Passive Shock Avoidance Paradigm

Lead-induced memory impairment was assessed using a passive shock avoidance test apparatus (PACS-30; Columbus Instruments, United States) that consists of two vertical compartments (23 cm^3^ × 24 cm^3^ × 24 cm^3^), with light and dark illumination separated by a guillotine door. On the 1st day, the animals were acclimatized with the compartments for 10 min to make animals familiar with the light and dark compartments. On the 2nd day, the rats were placed in a light compartment for 30 s of exploration time and the door was elevated and the time taken for the animal to go into the dark compartment was recorded for the next 5 min. An inescapable shock of 0.3 mA was given at the entry of the animal in the dark compartment. The memory of the shock given was retained by the animal, which was confirmed by conducting the same experiment but without shock and recording the latency time of the rat to go to the dark compartment. The trials were performed for the next 2 days, and shock was not provided during the acquisition phase (Leger et al., 2013).

### Sperm Parameters

The sperm motility, sperm count, sperm morphology, and sperm mitochondrial membrane potential were measured as described below.

#### Sperm Count and Motility

The sperm counts were measured as described by Babaknejad et al. (2018). The counting of the sperms was done under an optical microscope using a Neubauer hemocytometer. The sperm counts were expressed as (×10^6^ cells/ml of sperm) (Babaknejad et al., 2018).

The sperm motility was determined as described by Seed et al. (1996). The number of motile and immotile sperms was counted after mixing sperm samples with sodium citrate dehydrate, and the data were expressed as (%) (Seed et al., 1996).

#### Sperm Morphology

The sperm morphology was assessed using eosin stain as per the method described by Babaknejad et al. (2018). The sperm samples were mixed with eosin stain (kept for incubation) and spread out on a microscopic slide. The smears were prepared and observed under the microscope to visualize morphological changes.

#### Sperm Mitochondrial Membrane Potential (MMP)

The MMP in sperm samples was determined using JC-1 stain (lipophilic, cationic fluorescent dye). Briefly, 50 μl of epididymal sperm sample was mixed with JC-1 stain (1 μl), followed by incubation for 30 min. The smears were prepared and observed under an epifluorescence microscope. The yellow to orange fluorescence indicated active mitochondria, whereas green fluorescence indicated inactive mitochondria (Guvvala et al., 2017).

### Biochemical Variables

The various biochemical parameters were measured in the brain, spleen, and testes tissue samples as described below:

#### Superoxide Dismutase

The activity of superoxide dismutase (SOD) was measured as described by Kakkar et al. (1984) The reaction mixture consisted of 1.2 ml of sodium pyrophosphate, 0.3 ml of PMS, 0.3 ml of NBT, 0.2 ml of supernatant, 0.8 ml of distilled water, and 0.2 ml of NADH. The blank was prepared by adding distilled water in place of the sample. Both mixtures were incubated at 37°C for 90 s, and NADH was added at the last to initiate the reaction. The reaction was stopped by the addition of 1 ml acetic acid, and the mixture was cooled (for 10 min) at room temperature (Kakkar et al., 1984).

#### Reduced Glutathione

Glutathione (GSH) concentration was determined in the brain, spleen, and testes, as described by Gupta and Flora, (2005). Other proteins in the tissue homogenate were precipitated by adding an equal volume of 5% sulphosalicylic acid vortexed and kept in a resting position on an ice bath for 30 min. The supernatant from the centrifuged content was used for measuring GSH using Ellman’s reagent 5, 5-dithiobis (2-nitrobenzoic acid) (DTNB) solution. Results were expressed in μM GSH/mg protein (Ellman, 1961).

#### Catalase Activity

The catalase activity was determined according to the method described previously by Sinha (1972). The reaction mixture was prepared by incubating tissue homogenates with 0.5 ml of H_2_O_2_ (0.2 M) at 37°C for exactly 90 s in the presence of 0.01 M phosphate buffer (pH 7.4). The H_2_O_2_-initiated reaction was seized by adding 5% dichromate solution. All samples containing this mixture were incubated at 100°C for 15 min in a boiling water bath. Catalase activity was shown by the amount of H_2_O_2_ consumed, which was recorded by measuring the absorbance at 570 nm.

#### Thiobarbituric Acid Reactive Substances (TBARS)

Thiobarbituric acid is a measure of lipid peroxidation in tissue samples, which was performed as per the protocols suggested by Ohkawa et al. (1979). 100 μl tissue homogenate was mixed with 750 μl of 0.65% thiobarbituric acid, 100 μl sodium dodecyl sulfate (SDS), and 750 μl acetic acid. The reaction mixture for all samples was incubated in a boiling water bath, which formed a red complex of thiobarbituric acid–malondialdehyde (MDA) after 15 min, and the absorbance was recorded at 535 nm.

#### Reactive Oxygen Species

The measurement of reactive oxygen species (ROS) in tissue samples was carried out as per the protocols of Socci et al. (1999) *.* 10 μl of the supernatant from the tissue homogenate was diluted with 985 μl phosphate buffer with the addition of 5 μl DCFDA and incubated at 37°C for 30 min. A fluorescence plate reader was used to read the fluorescence intensity at 485 nm excitation and 529 nm emissions.

#### AChE Activity

Brain AChE activity was performed as per the protocols suggested by Mishra and Goel (2013). Briefly, 10 μl of brain homogenate was mixed with 95 μl phosphate buffer, which was then added to a reaction mixture of 4.75 ml phosphate buffer (pH 7.4), 0.315 ml DTNB, and 0.125 ml acetylthiocholine iodide (ATCI) of 104 μl in 96-well plates and shaken properly (Mishra and Goel, 2013).

#### Blood δ-aminolevulinic Acid Dehydratase

The activity of blood δ-aminolevulinic acid dehydratase (ALAD) was assayed according to the procedure of Flora et al. (2012). The assay consisted of 120 μl of heparinized blood and 540 μl of distilled water. After 10 min of incubation at 37°C for complete hemolysis, 420 μl of standard δ-aminolevulinic acid was added to the tubes and incubated for 60 min at 37°C. The reaction was stopped after 1 h by adding 420 μl of trichloroacetic acid. To the supernatant, an equal volume of Ehrlich’s reagent was added, and the absorbance was recorded at 555 nm after 5 min (Flora et al., 2012).

#### Protein Level

The protein level in the tissue samples was measured using Lowry’s method (Lowry et al., 1951). Supernatants (5 μl) of the tissue homogenates were incubated with solution D (2% sod. carbonate, 0.4% sod. hydroxide, 2% sod. tartrate, and 1% copper sulfate) for 10 min at 37°C. The resulting solution was treated with Folin’s reagent in a 1:1 ratio for 30 min at 37°C. The absorbance was measured at 660 nm along with a standard prepared with a known concentration of BSA.

### Immunological Variables

The delayed-type hypersensitivity reaction in rats was determined as footpad swelling. Pre-exposed rats were sensitized with 100 μl of bovine serum albumin (1 mg/ml) in Freund’s complete adjuvant (BSA-FCA) subcutaneously at the base of the tail. Rats from the normal control group were also sensitized as described above. Later, after 7 days, the challenge involved 25 μl of a 2% (w/v) solution of BSA injected at the left footpad. The right footpad was injected with saline. On day 8 (24 h later), both footpads were swollen, which was measured using a vernier caliper (Scienceware^®^ vernier caliper, direct-reading type). The data were represented as the mean difference in swelling (in mm) between footpads (Flora and Kumar, 1996).

#### Measurement of Tissue Caspase-3, Cytokine Profiles, and Serum Immunoglobulins

The secretory levels of cytokines IL-4, IL-6, IL-1β, and TNF-α in the rat’s brain and spleen were quantitatively determined using ELISA kits (ELK Biotechnology, BOSTER, Wuhan, China) according to the manufacturer’s instructions (Catalog no. IgE: ELK2594, IgM: ELK1349, caspase-3: ELK1528, IgA: ELK2596, IgG: ELK1393, IL-4: ELK1154, IL-1β: ELK1272, IL-6: ELK1158, and TNF-α: ELK1396). Immunoglobulins IgM, IgG, IgA, and IgE were also determined using ELISA commercial kits (ELK Biotechnology, BOSTER, Wuhan, China) in rat serum samples. The results were compared with the results obtained in the calibration curve (*R* ^2^ > 0.998).

### Elemental Analysis

Measurement of blood metal ion concentration (lead and zinc) was done using inductively coupled plasma mass spectrometry (ICP-MS). The blood metal digestion procedure involves overnight incubation of blood with 3 ml of nitric acid and 0.2 ml perchloric acid in a 3:1 ratio. Later, it was heated repetitively by adding this acid mixture until we got a white solid mass. The solid mass was dissolved in warm 0.5% nitric acid and was filtered using the Whatman filter paper. Triple distilled water was used throughout, and the final volume of the filtrate was made up to 10 ml (Patwa et al., 2020).

### Histopathological Analysis

The dissected testis and brain tissues were washed in chilled PBS and then fixed in a 10% buffer formalin solution. The hippocampus and cortex were separated from the whole brain samples. The fixed tissue samples were processed in different grades of alcohol and xylene and were embedded in paraffin blocks. Sections (5 μm) were cut from paraffin blocks using a microtome, stained with hematoxylin and eosin (H&E), and mounted with DPX. Slides were observed under a light microscope to examine gross cellular damage and histological alterations. The hippocampus was observed for different areas such as CA1, CA2, CA3, and DG regions (Shaibah et al., 2016) (Navaie et al., 2018).

### Statistical Analysis

The statistical analysis was done using GraphPad Prism version 6.0. The results were presented as mean ± standard error of mean (SEM). All statistical comparisons were performed by using means of one-way analysis of variance (ANOVA) followed by Tukey’s multiple comparison post hoc tests, and *p*-value less than 0.05 was considered to be significant.

## Results

### Characterization of Zinc Oxide Nanoparticles by Transmission Electron Microscopy (TEM), Dynamic Light Scattering, and Zeta Potential Measurement

**FIGURE 2:**
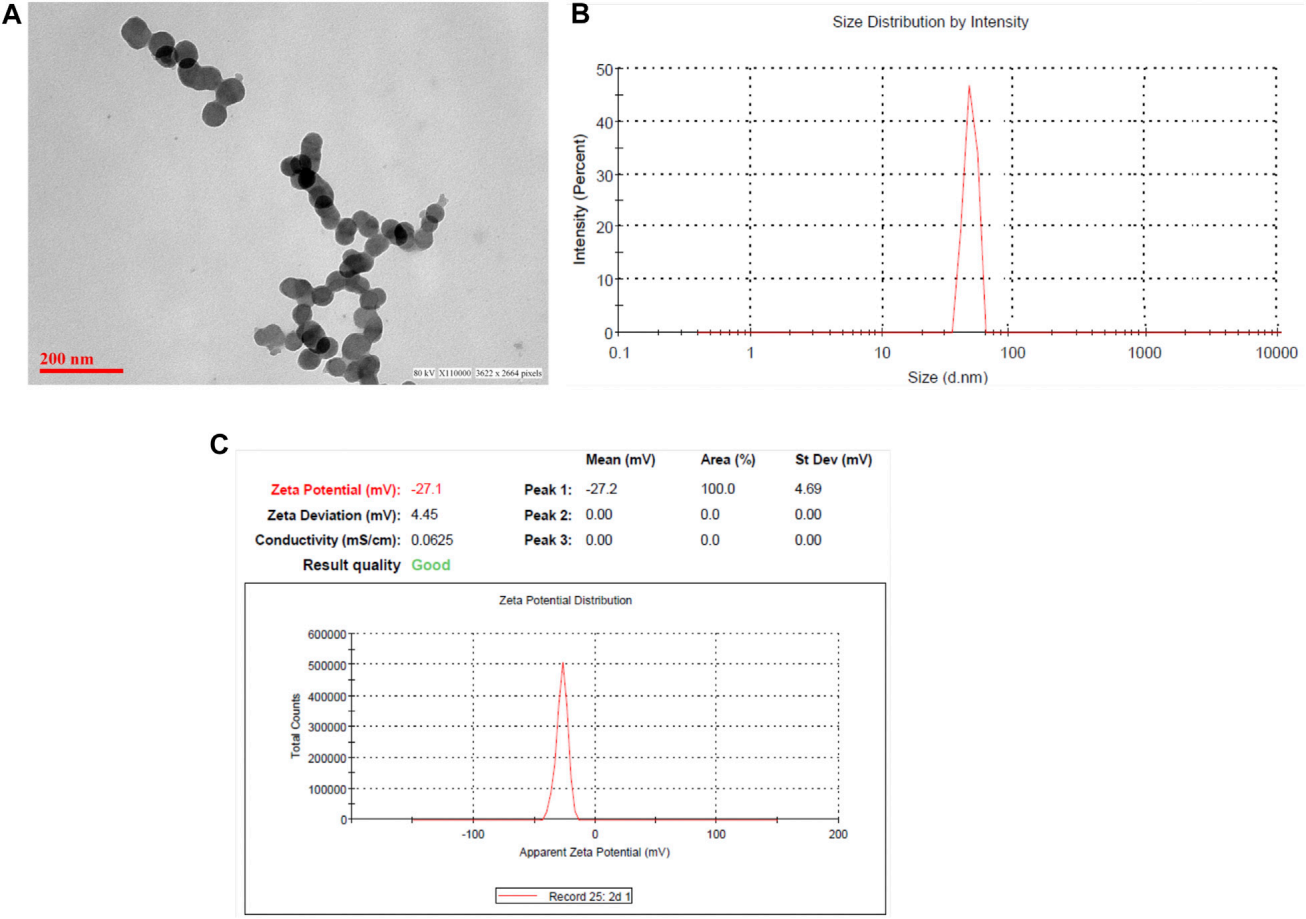
Characterization of zinc oxide nanoparticles. **(A)** Transmission electron microscopy of zinc oxide nanoparticles; **(B)** the hydrodynamic size of NPs determined by dynamic light scattering (DLS) studies; and **(C)** zeta potential.

The particle size, size distribution, and zeta potential measurements of the ZnO NPs were conducted using a Zetasizer as shown in Figure 2. The size and shape of the zinc oxide nanoparticles were analyzed by transmission electron microscopy (TEM). Figure 2A demonstrates that zinc oxide is spherical and monodispersed in nature, with an average particle size of around 60 nm. The average hydrodynamic diameter of ZnO NPs was around 60–70 nm (Figure 2B). The ZnO NPs exhibited a negative charge, that is, −27.2, which was confirmed by the zeta potential results of the ZnO NPs (Figure 2C).

### Effects on Neurobehavioral Parameters

#### Spontaneous Locomotor Activity

**FIGURE 3:**
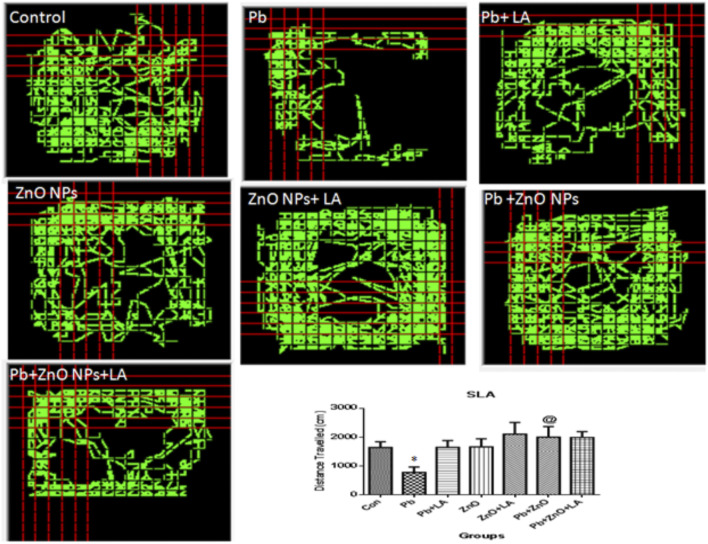
Locomotor activity in rats using the Optovarimax apparatus. All the values are expressed as mean ± SEM (n=5). ^*^ *p* < 0.05 vs. control; ^@^ *p* < 0.05 vs. lead.

The spontaneous activity and exploration were evaluated in the open-field test. The data for spontaneous locomotor activity suggested that the locomotor activity of α-lipoic acid–treated rats was increased in comparison to other groups (Figure 3).

#### Effect on Transfer Latency in the Passive Shock Avoidance Test

**FIGURE 4:**
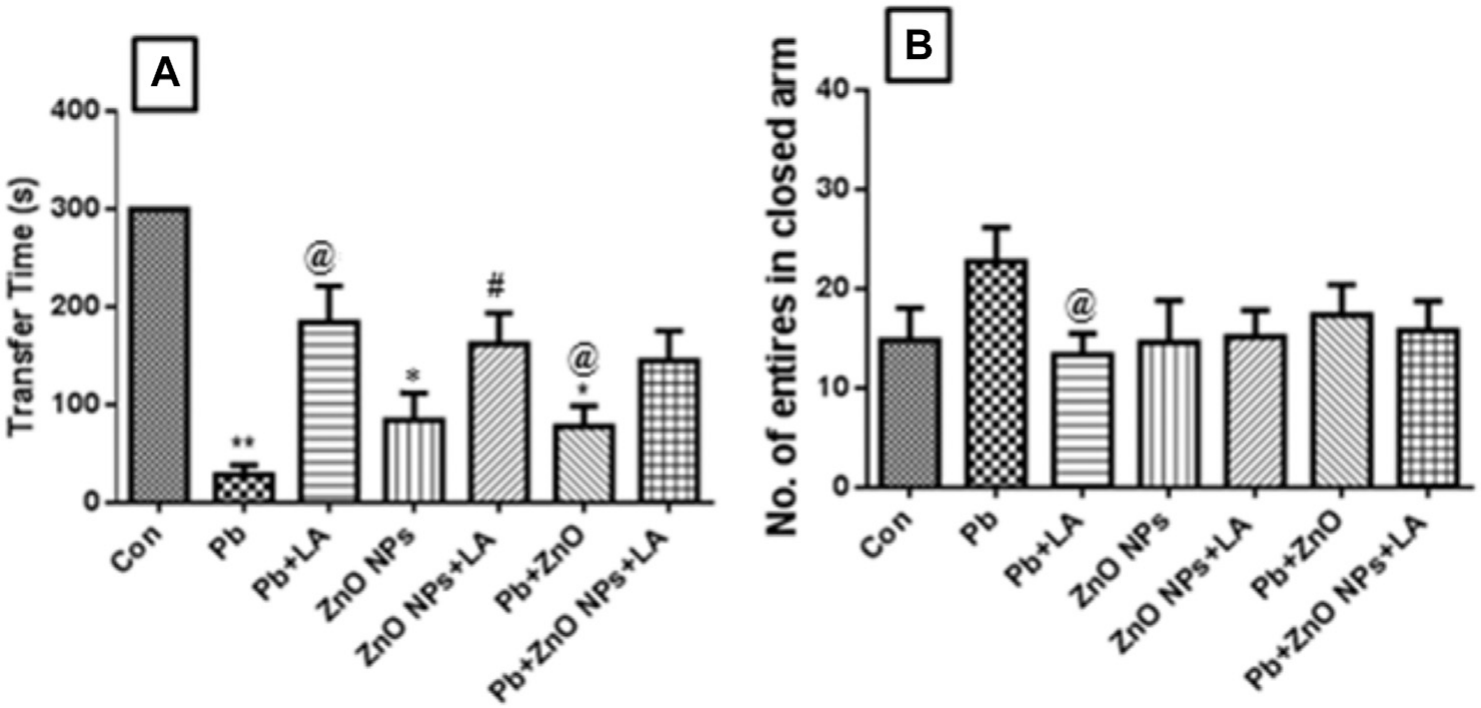
Latency of transfer to the dark compartment **(A)**. Number of entries in the closed arm **(B)**. All the values are expressed as mean ± SEM (n = 5). *p < 0.05, **p < 0.01 vs. control; @*p* < 0.05 vs. lead; #*p* < 0.05 vs. ZnO NPs.

Lead alone and in combination with ZnO NPs–treated group animals exhibited a significant difference in the memory performance compared to the control. The passive avoidance test showed the latency time taken to go into the compartment where the shock was given in a habituation phase (Figure 4A).

#### Elevated Plus-Maze (EPM) Assessment

EPM results demonstrate that lead-treated rats showed more number of entries in a closed arm. The numbers of entries in a closed arm were indicative of fear and anxiety in the animals. The results were not significant, but compared to α-lipoic treatment, it showed a high number of entries in a closed arm (Figure 4B).

### Effects on Reproductive Parameters

#### Testes and Epididymis Weight

**FIGURE 5:**
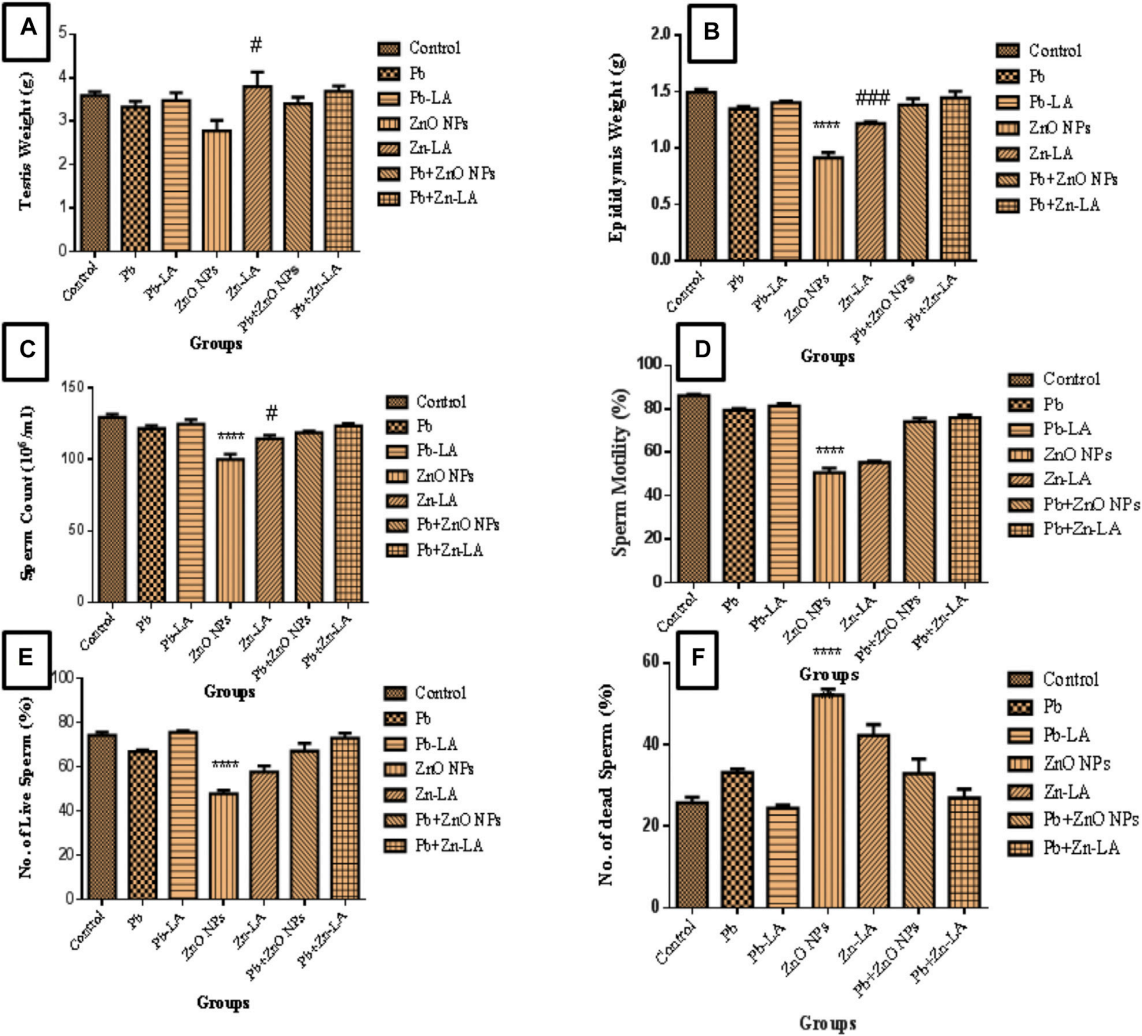
Effect on the weight of **(A)** testes, **(B)** epididymis, **(C)** sperm count, **(D)** sperm motility, **(E)** a number of live sperms (%), and **(F)** a number of dead sperms (mean ± SEM). Statistical significance is represented as ****p <0.0001 as compared with control and ^#^ *p* <0.05, ^###^ *p* <0.001 as compared with zinc oxide nanoparticles alone group.

There was no significant change in testes weight in all groups of animals, except in the zinc oxide nanoparticle and lipoic acid groups (Figure 5A). The weight of the epididymis was significantly decreased in the zinc oxide nanoparticle group compared to the control. The lipoic acid treatment increased epididymis weight, which was decreased by zinc oxide nanoparticles (Figure 5B).

#### Sperm Analysis

The sperms were analyzed for sperm count, motility, morphology, and mitochondrial membrane potential (MMP) to check the effect of lipoic acid against lead-altered and ZnO NP–altered male reproductive parameters.

##### Sperm Count and Motility

The sperm count and sperm motility were significantly decreased in the zinc oxide nanoparticle group compared to the control. However, there was a nonsignificant decrease in the lead alone and lead plus zinc oxide nanoparticle groups. The decreased sperm counts and motility by zinc oxide nanoparticles were significantly increased by lipoic acid treatment (Figures 5C,D).

##### Sperm Morphology

**FIGURE 6:**
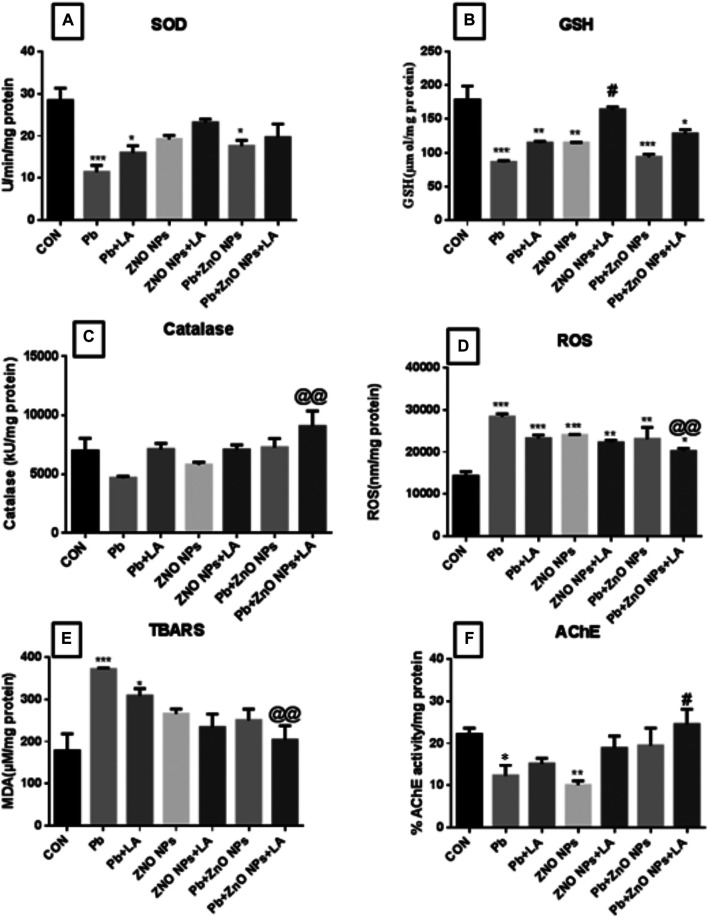
Brain biochemical parameters and AChE activity. **(A)** SOD activity; **(B)** GSH activity; **(C)** catalase activity; **(D)** ROS level; **(E)** TBARS level; and **(F)** AChE activity. All the values are expressed as mean ± SEM (n = 4). The statistical significance was considered at *p* < 0.05, and statistical analysis was performed using one-way ANOVA, followed by Tukey’s test. *p < 0.05, **p < 0.01, ***p < 0.001 vs. Control; ^@@^ *p* < 0.01 vs. lead; ^#^ *p* < 0.05, ^##^ *p* < 0.01 vs. ZnO NPs. SOD: superoxide dismutase; GSH: glutathione; ROS: reactive oxygen species; TBARS: thiobarbituric acid reactive substances; AChE: acetylcholinesterase; SEM: standard error of mean; and ANOVA: analysis of variance.

The normal sperm morphology was observed in all the groups of animals, except the zinc oxide nanoparticle group (Figures 6A–G). The altered morphology by zinc oxide nanoparticles was normalized after lipoic acid treatment (Figure 6D).

##### Sperm Mitochondrial Membrane Potential

The percentage of live sperms were significantly decreased, whereas the percentage of dead sperms were significantly increased in the zinc oxide nanoparticle group compared to the control group. The MMP in the lead alone group and the combination with zinc oxide nanoparticle group showed depletion in a nonsignificant manner (Figures 5E,F). Treatment with lipoic acid resulted in a nonsignificant increase in the percentage of live sperm and a decrease in the percentage of dead sperms as compared to the zinc oxide nanoparticle group (Figure 5).

### Effects on Hematopoietic Variables and Blood Oxidative Stress

#### Variable Indicative of Alterations in Heme Synthesis

**FIGURE 7:**
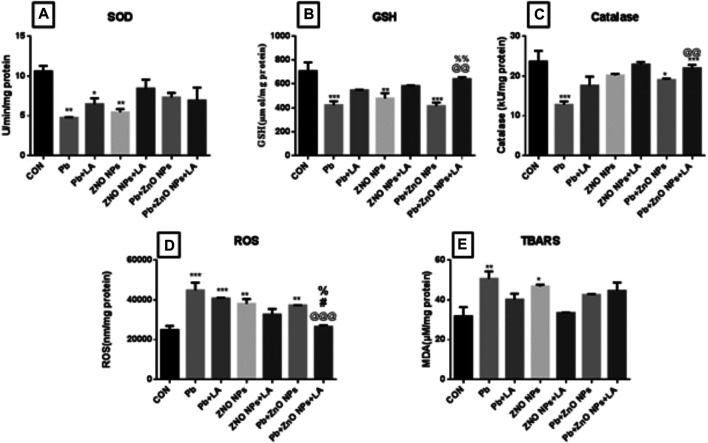
Spleen biochemical parameters. **(A)** SOD activity; **(B)** GSH activity; **(C)** catalase activity; **(D)** ROS level; and **(E)** TBARS level. All the values are expressed as mean ± SEM (n = 4). The statistical significance was considered at *p* < 0.05, and statistical analysis was performed using one-way ANOVA, followed by Tukey’s test. *p < 0.05, **p < 0.01, ***p < 0.001 vs. control; ^@@^ *p* < 0.01, ^@@@^ *p* < 0.001 vs. lead; ^#^ *p* < 0.05 vs. ZnO NPs; ^%^ *p* < 0.05, ^%%^ *p* < 0.01 vs. PB + ZnO NPs.

Lead inhibited blood ALAD activity, the enzyme involved in the heme biosynthesis pathway, in a dose-dependent manner. We also observed a significant fall in blood ALAD activity in the lead-, ZnO NP–, and Pb + ZnO NP–treated groups (Figure 7A). A moderate beneficial effect of LA was noted in the inhibited activity of blood ALAD activity in these groups.

#### Oxidative Stress Parameters in the Brain

**FIGURE 8:**
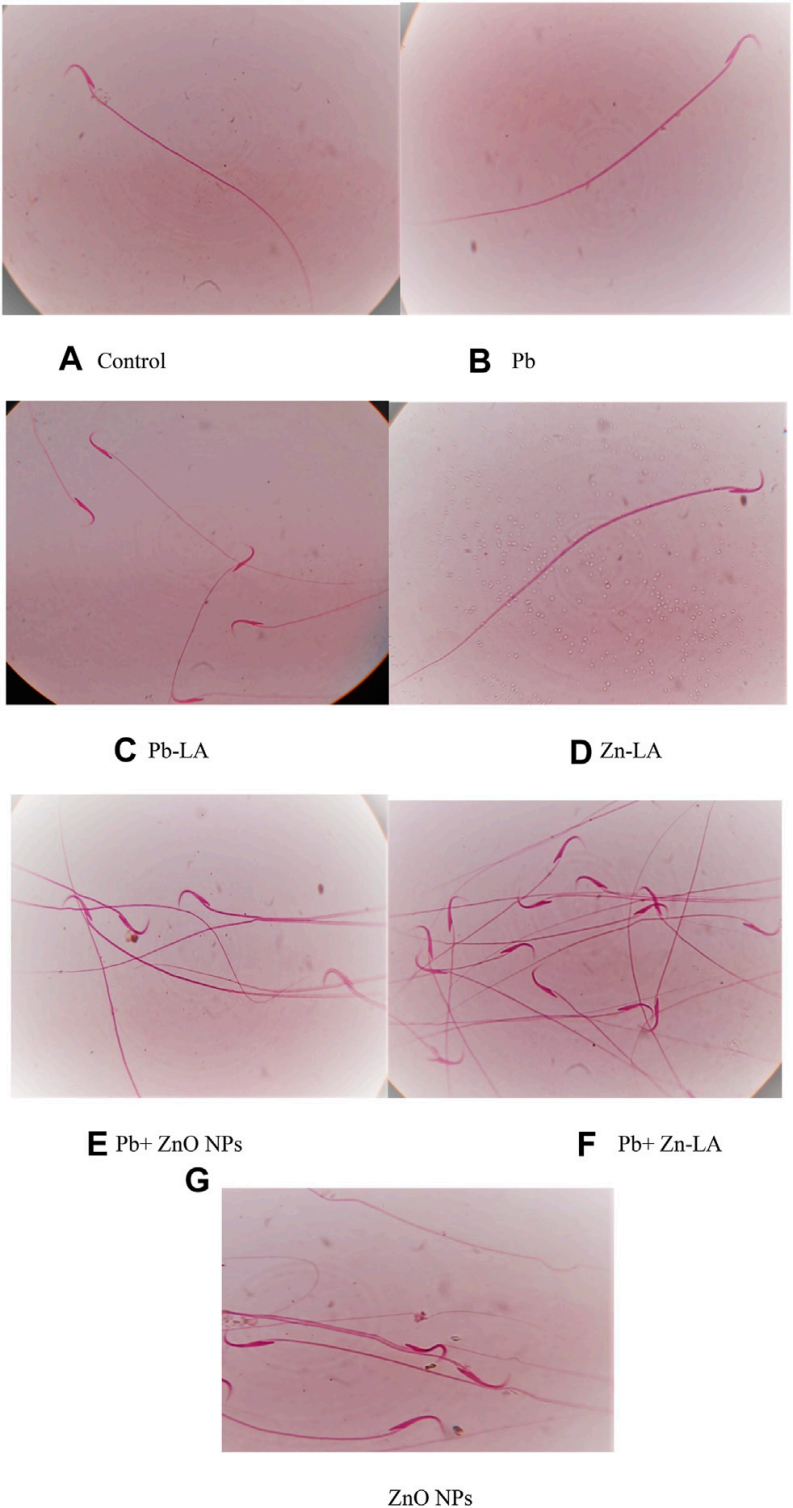
Effect of lipoic acid on lead- and zinc oxide nanoparticles–altered sperm morphology. The normal morphology of sperms was shown in figures **(A–F)**. The morphological abnormalities were indicated by arrows **(G)**.

GSH, SOD, and catalase are indicative of antioxidant status. There was a significant decrease in GSH activity in the lead- and ZnO NP–treated groups alone and in combination which was attenuated by α-lipoic acid treatment (Figure 8). There was a significant decrease in SOD activity in the lead- and ZnO NP–treated groups (Figure 8A). There was a significant increase in the ROS level in the lead- and ZnO NP–treated groups alone and in combination which was attenuated by α-lipoic acid treatment (Figure 8D). There was a significant increase in the TBARS level in the lead group, which was restored after α-lipoic acid treatment (Figure 8E). The AChE activity showed a significant increase in the combination group when treated with α-lipoic acid compared to the ZnO NP group (Figure 8F).

#### Oxidative Stress Parameters in the Spleen

**FIGURE 9:**
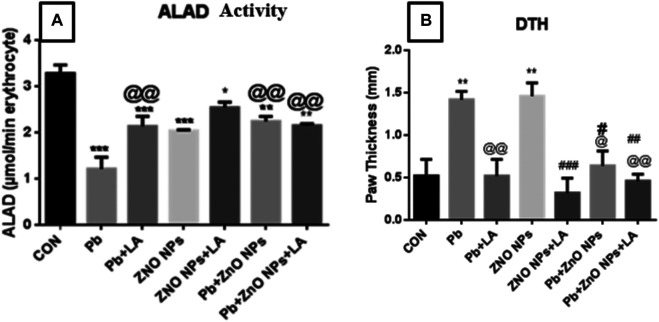
**(A)** Blood delta-aminolevulinic acid dehydratase activity. **(B)** Rat paw thickness (in mm). All the values are expressed as mean ± SEM (n = 5). The statistical significance was considered at *p* < 0.05, and statistical analysis was performed using one-way ANOVA, followed by Tukey’s test. *p < 0.05, **p < 0.01, ***p < 0.001 vs. control; @*p* < 0.05, @@*p* < 0.01 vs. lead; #*p* < 0.05, ##*p* < 0.01, ###*p* < 0.001 vs. ZnO NPs.

There was a significant decrease in GSH, SOD, and catalase activity in the lead- and ZnO NP–treated groups, which was attenuated by α-lipoic acid treatment (Figures 9A–C). A significant increase in lead- and ZnO NP–treated groups alone and in combination was attenuated by α-lipoic acid treatment (Figure 9D). There was also a significant increase in the TBARS level in lead and ZnO NP–exposed groups compared to the control (Figure 9E).

#### Oxidative Stress Parameters in Testes

**FIGURE 10:**
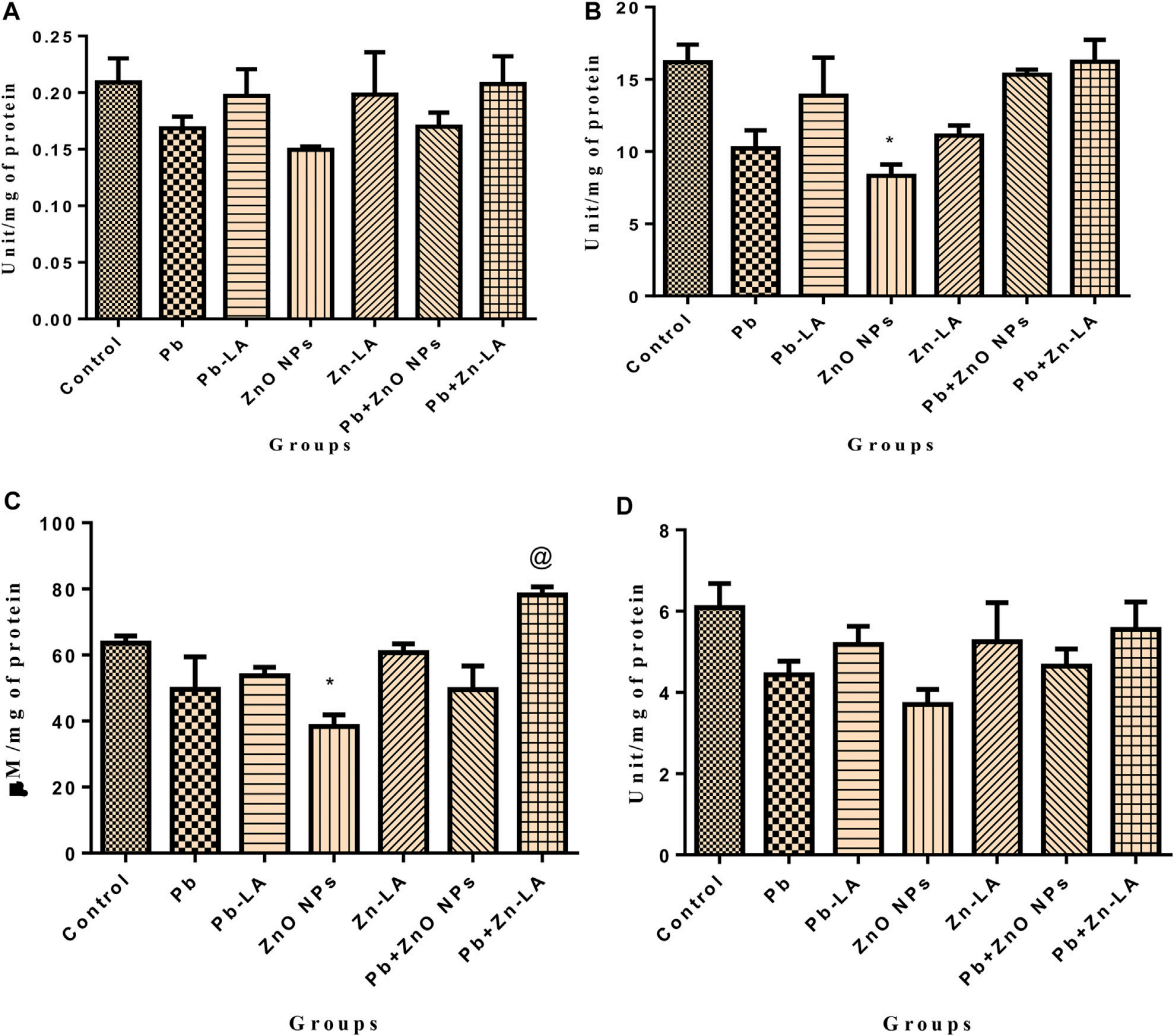
Testes biochemical parameters. **(A)** SOD, **(B)** catalase, **(C)** GSH, and **(D)** GPx. The data are presented as mean ± SEM. Statistical significance is represented as *p <0.05, as compared to the control group and @*p* <0.05, as compared to the combination group.

There was a nonsignificant change in the level of ROS in all groups of animals as shown in Figure 10A. However, the level of MDA increased significantly in the ZnO NP group compared to the control. The increased level of MDA was significantly decreased by lipoic acid treatment (Figure 10B).

The activity of catalase and the GSH level were significantly decreased in the zinc oxide nanoparticle group compared to the control group (Figures 10B,C). However, SOD and GPx activities in all the groups did not change significantly (Figures 10A,D). Treatment with lipoic acid increased the level of antioxidant enzymes (Figure 10).

#### Effects on Immunological Variables

Cell-mediated immune function was assessed using delayed-type hypersensitivity response. There was a significant increase in the lead- and ZnO NP–treated groups alone and in combination which was attenuated by α-lipoic acid treatment (Figure 7B).

##### Effects on Serum Immunoglobulins

**FIGURE 11:**
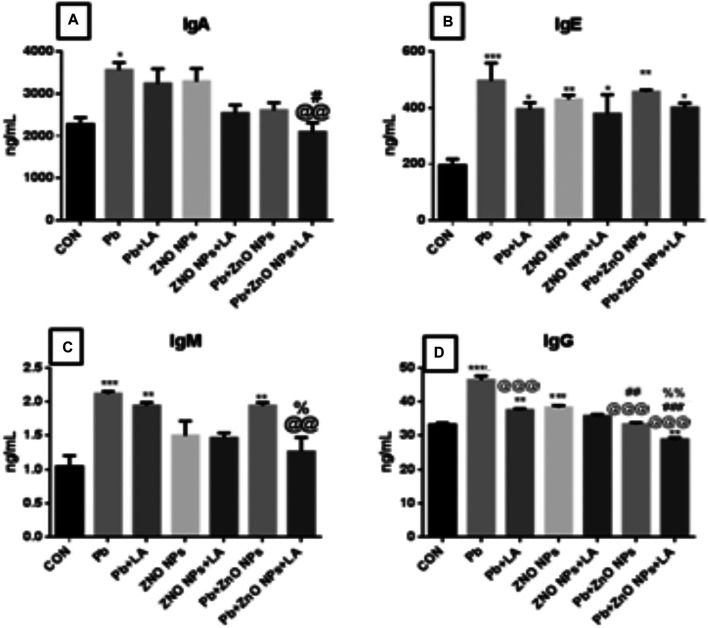
Levels of immunoglobulins in rat serum. **(A)** IgA; **(B)** IgE; **(C)** IgM; and **(D)** IgG. All the values are expressed as mean ± SEM (n = 4). The statistical significance was considered at *p* < 0.05, and statistical analysis was performed using one-way ANOVA, followed by Tukey’s test. ^*^ *p* < 0.05, ^**^ *p* < 0.01, ^***^ *p* < 0.001 vs. control; ^@@^ *p* < 0.0, ^@@@^ *p* < 0.01 vs. lead; ^#^ *p* < 0.05, ^##^ *p* < 0.01 vs. ZnO NPs; ^%^ *p* < 0.05, ^%%^ *p* < 0.01 vs. PB + ZnO NPs. Ig: immunoglobulin.

There was a significant increase in IgA (*p* < 0.05), IgE, and IgM (*p* < 0.001) levels in lead- and ZnO NP–treated groups, respectively (Figures 11A-C). IgG levels were significantly increased in the lead- and ZnO NP–treated groups alone and in combination which was attenuated by α-lipoic acid treatment (Figure 11D).

##### Cytokines and Caspase-3 in the Brain

**FIGURE 12:**
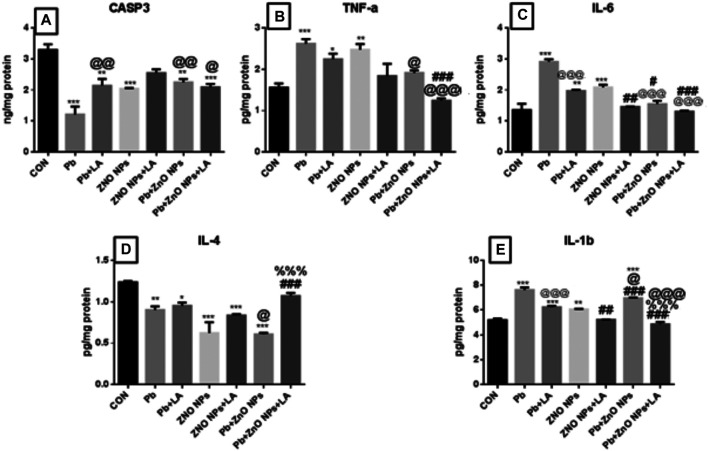
Rat brain caspase-3 and cytokine levels. **(A)** CASP3; **(B)** TNFa; **(C)** IL-6; **(D)** IL-4; and **(E)** IL-1b. All the values are expressed as mean ± SEM (n = 4). The statistical significance was considered at *p* < 0.05 and statistical analysis was performed using one-way ANOVA, followed by Tukey’s test. ^*^ *p* < 0.05, ^**^ *p* < 0.01, ^***^ *p* < 0.001 vs. control; ^@^ *p* < 0.05, ^@@^ *p* < 0.01,^@@@^ *p* < 0.001 vs. lead; ^#^ *p* < 0.05, ^##^ *p* < 0.01,^###^ *p* < 0.001 vs. ZnO NPs; ^%%%^ *p* < 0.001 vs. lead+ ZnO NPs. CASP: caspase; TNF: tumor necrosis factor alpha; IL: interleukin; SEM: standard error of mean; and ANOVA: analysis of variance.

There was a significant increase in caspase-3, TNF, IL-1, and IL-6 in lead- and ZnO NP–treated groups alone and in combination, which was attenuated by α-lipoic acid treatment (Figure 12 resp.) All α-lipoic acid–treated groups showed significant reversal. There was a significant decrease in the IL-4 level in the lead- and ZnO NP–treated groups alone and in combination which was attenuated by α-lipoic acid treatment (Figure 12).

##### Cytokines and Caspase-3 in the Spleen

**FIGURE 13:**
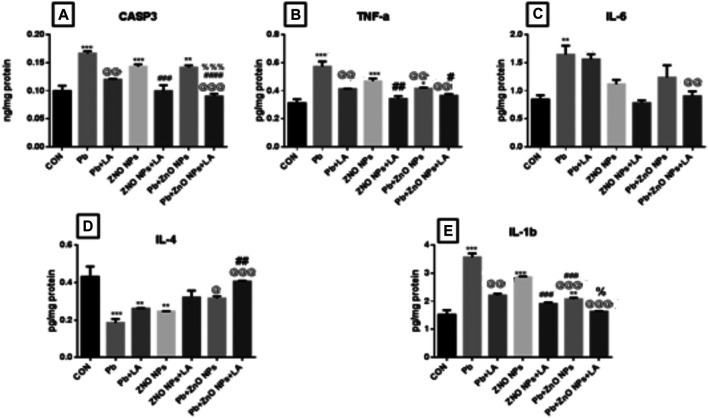
Rat spleen caspase-3 and cytokine levels: **(A)** CASP3; **(B)** TNFa; **(C)** IL-6; **(D)** IL-4; and **(E)** IL-1b. All the values are expressed as mean ± SEM (n = 4). The statistical significance was considered at *p* < 0.05, and statistical analysis was performed using one-way ANOVA, followed by Tukey’s test. ^*^ *p* < 0.05, ^**^ *p* < 0.01, ^***^ *p* < 0.001 vs. control; ^@^ *p* < 0.05, ^@@^ *p* < 0.01, ^@@@^ *p* < 0.001 vs. lead; ^#^ *p* < 0.05, ^##^ *p* < 0.01,^###^ *p* < 0.001 vs. ZnO NPs; ^%^ *p* < 0.05, ^%%%^ *p* < 0.001 vs. PB + ZnO NPs. CASP: caspase; tNF: Tumor necrosis factor alpha; IL: interleukin; SEM: standard error of mean; and ANOVA: analysis of variance.

The rat spleen showed a significant increase in caspase-3, TNF, IL-1, and IL-6 in lead- and ZnO NP–treated groups alone and in combination which was significantly protected by α-lipoic acid treatment (Figure 13 resp.) All α-lipoic acid–treated groups showed significant recovery. There was a significant decrease in the IL-4 level in lead- and ZnO NP–treated groups both during individual exposure and in combination, which was significantly protected by α-lipoic acid treatment (Figure 13).

#### Metal Levels in Blood

A significant increase in the level of lead concentration in lead acetate–treated groups was significantly reduced on α-lipoic acid treatment (Table 2). A significant decrease and an increase in zinc levels were observed in the lead- and zinc oxide–treated groups, respectively (Table 2).

### Histopathological Observations

**FIGURE 14:**
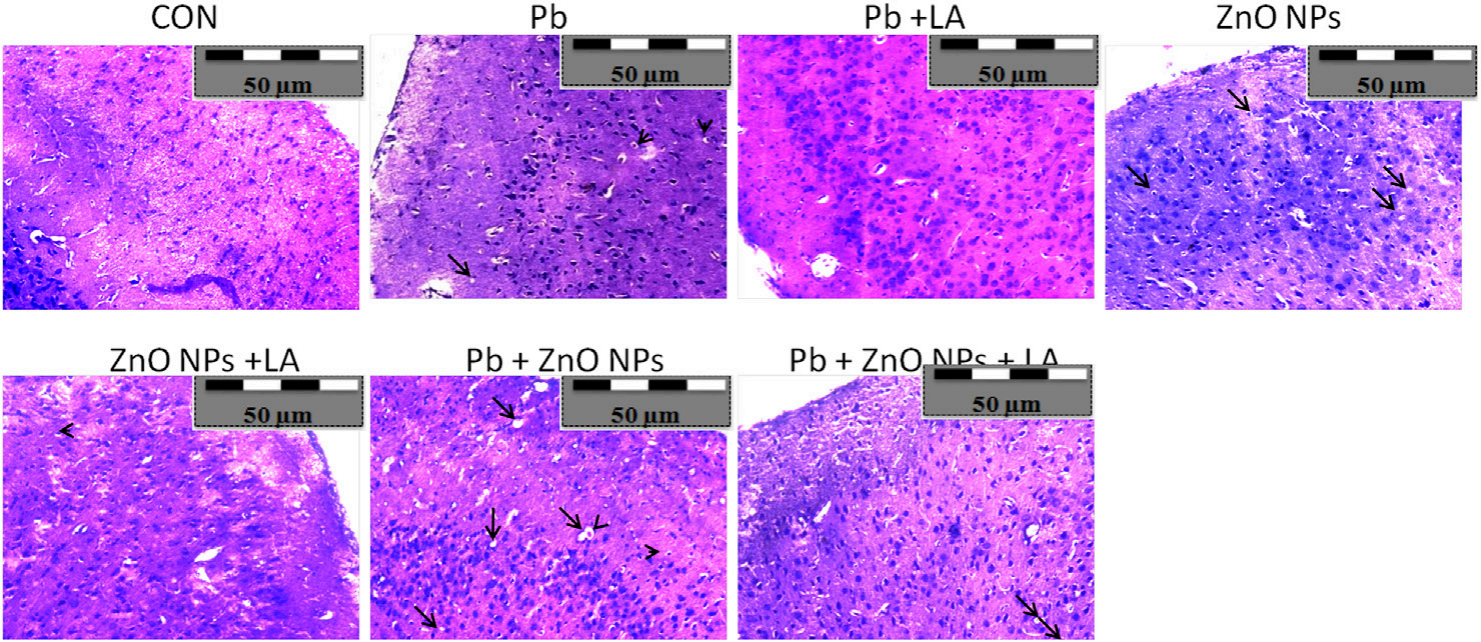
Photographs of histological sections of the cerebral cortex of rats showing **(A)** hyperchromatic cells (arrow-head) and **(B)** pyknosis (arrow).

**FIGURE 15:**
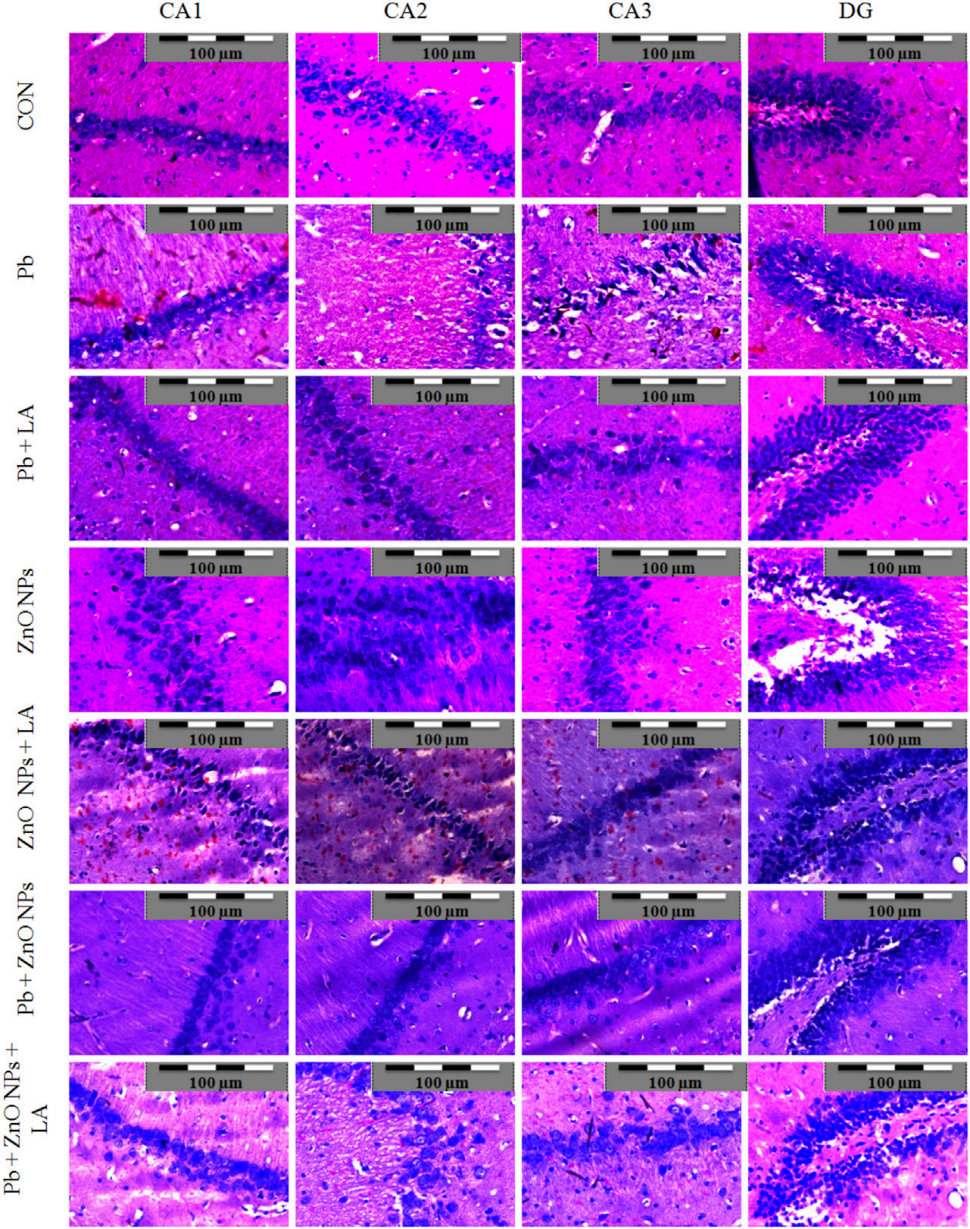
Photomicrographs of the hippocampus of experimental animals. Lead- and ZnO NP–treated groups showing altered cell density in the CA1, CA2, CA3, and DG regions.

**FIGURE 16:**
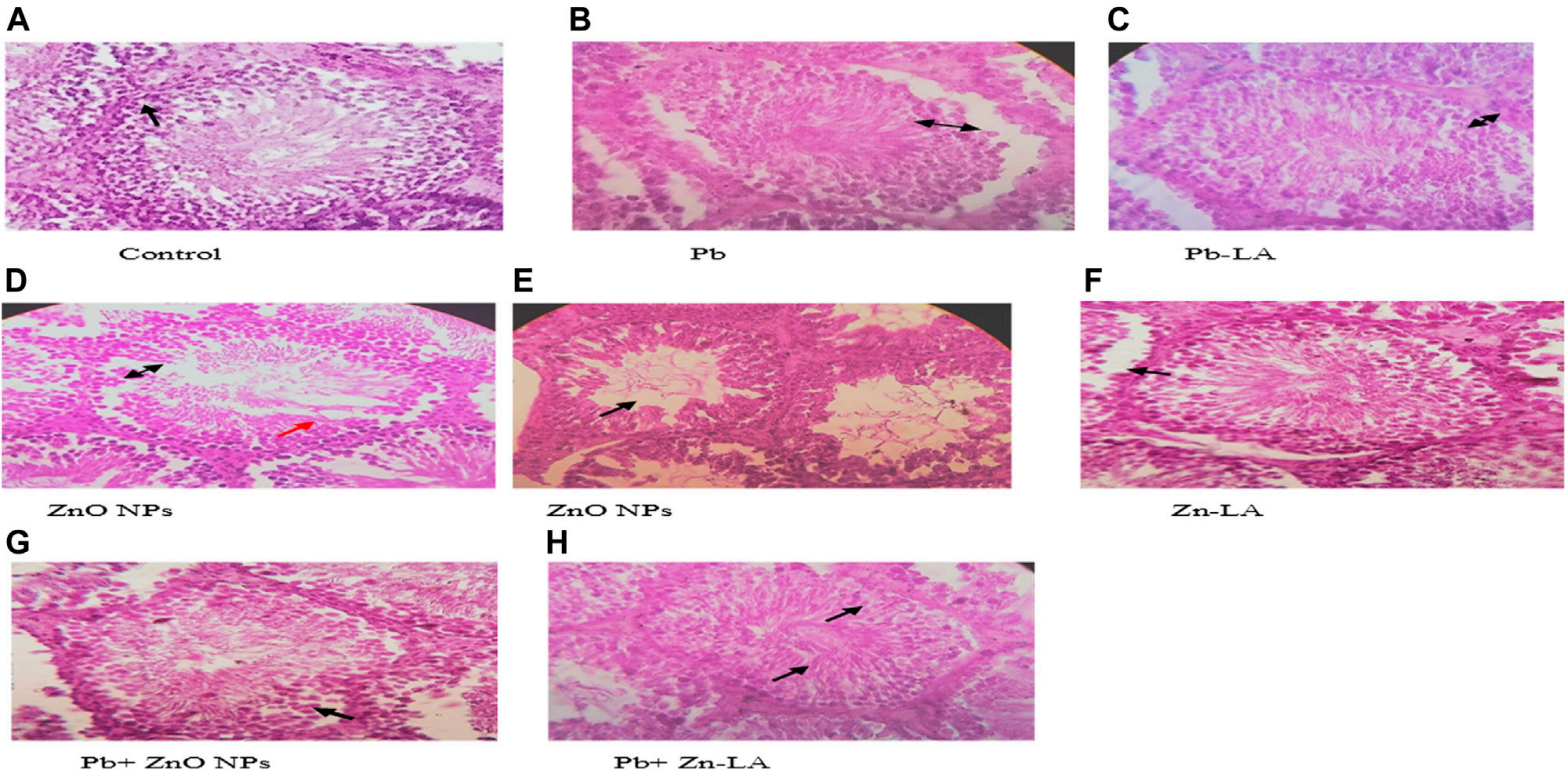
Photographs of the testes of experimental animals. **(A)** Control group showing normal seminiferous tubules (Sts) lined by stratified germinal epithelium (as presented by black arrow). **(B)** Lead-treated group showing separated germinal epithelium from their basement membrane (black arrow). **(C)** lipoic acid–treated lead group showing a regular basal lamina (black arrow). **(D)** Zinc oxide nanoparticle group showing degeneration of germinal epithelium (black arrow) and vacuolations (red arrow). **(E)** Zinc oxide nanoparticle group showing the large diameter of lumen (black arrow). **(F)** Lipoic acid–treated zinc oxide nanoparticle group showing a regular basal lamina and germinal epithelium layer (black arrow). **(G)** Lead and zinc oxide nanoparticle groups showing irregular basal lamina (black arrow). **(H)** Lipoic acid–treated lead and zinc oxide nanoparticle groups showing a regular basal lamina, germinal epithelium layer, and sperms in the lumen (black arrows).

Histological sections of the rat cerebral cortex and hippocampus stained with hematoxylin and eosin were observed below a light microscope. Brain sections of control animals showed normal histological architecture, whereas the lead- and ZnO NPs–treated groups showed hyperchromatic cells and pyknosis (Figure 14). Comparison of α-lipoic acid–treated groups with their respective controls showed its protective effect. The hippocampus was observed for cell density in the CA1, CA2, CA3, and DG (Dentus gyrus) subfields. Both the lead- and ZnO NP–treated groups showed altered cell density, which was not found in α-lipoic acid–treated groups (Figure 15). The histopathological studies of testis samples (control, Pb-LA, Zn-LA, and Pb + Zn-LA groups) revealed a normal testicular shape and morphology with spermatogenic cells (Figure 16). The alterations in the seminiferous tubules such as reductions in the number of germ cells in spermatogenesis, degeneration, vacuolization, and large lumen of seminiferous epithelium were observed in the lead alone (Figure 16B), zinc oxide nanoparticles alone (Figure 16D), and in combination groups (Figure 16G). The lipoic acid treatment attenuated the lead- and zinc oxide nanoparticles–induced structural changes in the testes (Figures 16C,F,H).

## Discussion

It has been reported that excess exposure to ZnO NPs and lead results in multi-organ dysfunction. However, the effects of these metals in combination have rarely been studied and are largely unclear. Since the toxic effects of combined exposure remain a mystery, the treatment approaches also remain unclear. The present study highlights the role of lipoic acid against combined lead and ZnO NP exposure along with its effect on individual exposure to these toxicants in male Wistar rats.

The nervous system is one of the most sensitive systems against metal-induced toxicity. The impairment of learning and memory in lead-exposed animals has been widely studied and reported (Jan et al., 2015). The mechanism involved includes permeability of the blood–brain barrier (BBB), ability to replace calcium, and damage to brain areas involving the cerebral cortex, hippocampus, and cerebellum. (Sanders et al., 2009). Lead also interferes with the release of neurotransmitters, disrupting GABAergic function and dopaminergic and cholinergic systems along with inhibiting NMDA-ion channels during the neonatal period (Sharma et al., 2015). Earlier studies have reported the inhibition of acetylcholinesterase activity on lead and ZnO NP exposure (Nehru and Sidhu, 2002; Reddy et al., 2003). The levels were particularly observed in brain areas like the cortex, hippocampus, and cerebellum, mainly responsible for memory function. We also observed a decreased level of brain AChE in lead- and ZnO NP–exposed groups. Further, treatment with lipoic acid restored these effects to a moderate level. Lead has a binding affinity toward sulfur and oxygen atoms in proteins, leading to oxidative stress (Jan et al., 2015; Kurutas, 2016). Zinc oxide nanoparticles, on the other hand, are known to play a largely protective role but exhibit toxicity depending upon the dosage, duration of exposure, etc. We noted the beneficial effects of alpha-lipoic acid treatment on lead and ZnO NP–induced behavioral alteration in rats.

The immune system is one of the complex systems of the body, which protects us against a variety of pathogens. When a person encounters a pathogen, the body’s immune system develops immunity particularly against the pathogen in addition to the other protection mechanisms. The specific immune system employs two classes of cells: B cells and T cells. B cells are precursors of antibody-secreting plasma cells which play a role in humoral immunity and produce five major classes of immunoglobulin molecules. Meanwhile, T cells involved in the cell-mediated immunity include an array of subtypes of cells that mediates immunoregulatory functions by mechanisms such as producing lymphokines and direct destruction of antigen-bearing cells (on Descotes, 2006). Cytokines perform pleiotropic functions to mediate and regulate the immune response and are thus recognized as biomarkers of immunotoxicity (Elsabahy and Wooley, 2013). Lead affects the immune functions by altering the cytokine production, which is involved in the inflammatory processes (Kurutas, 2016). Furthermore, the inflammatory and other immune pathologies altered by lead contribute toward other CNS-related pathologies, particularly, psychiatric disorders. The role of neurodegeneration in neurological disorders like Alzheimer’s disease (AD), Parkinson’s disease (PD), or multiple sclerosis (MS) and their association with the immune system are still under investigation. The activated immune system disturbs pro-inflammatory and anti-inflammatory responses, both centrally and peripherally. A peripheral immune mediator such as cytokines enters the CNS *via* the BBB and causes chronic neuro-inflammation and damages after sustained release (Amor et al., 2010). The toxic effects of lead on the immune system are variable (Fenga et al., 2017). Treatment with alpha-lipoic acid decreased both lead- and ZnO NPs–altered inflammatory markers in rats in our study.

Blood lead levels (BLLs) and δ-aminolevulinic acid dehydratase (ALAD) activity are considered as the early and specific biomarkers of lead exposure and toxicity, respectively. Delta-aminolevulinic acid dehydratase (δ-ALAD) is a cytosolic enzyme that catalyzes the formation of porphobilinogen from δ-aminolevulinic acid (ALA), and a significant alteration in the heme synthesis occurs on exposure to lead by the downregulation of the delta-aminolevulinic acid dehydratase (ALAD) enzyme (La-Llave-León et al., 2017). We also observed significant inhibition of blood δ-ALAD in lead and ZnO NP groups and a positive response after treatment with α-lipoic acid. Lead concentration too showed a significant response but only a moderate decrease in the animals treated with α-lipoic acid. On the other hand, blood zinc levels significantly decreased in animals coexposed to lead and ZnO NP. This could be due to the mimicking property of zinc, the overutilization of zinc by the enzymes, and the fact that zinc and lead fight for the same binding site *in vivo*. The antagonistic effects of zinc against lead are well known (El-Gazzar et al., 1978).

Male fertility is one of the major problems over the past few decades. Recently, we also reported testicular toxicity in rats after chronic exposure to multi-metals (Gupta et al., 2021). Various reports have indicated toxic effects of lead and zinc oxide nanoparticles on reproductive organs in experimental animals depending upon dose and duration (Lee et al., 2016; Abbasalipourkabir et al., 2015; Batra et al., 1998). They reported that sperm counts and motility were significantly decreased after zinc oxide nanoparticle exposure. This might be due to zinc oxide nanoparticle–induced oxidative stress. Toxic effects of zinc oxide nanoparticles and lead on the testis and epididymis have been reported earlier (Abbasalipourkabir et al., 2015; Talebi et al., 2013; Hari Priya and Reddy, 2012). Lipoic acid attenuated zinc oxide nanoparticle–altered sperm count and motility. Mitochondrial membrane potential (MMP) is an essential component in the process of energy storage during oxidative phosphorylation and helps in the movement of the sperm. The zinc oxide nanoparticle exposure resulted in a decreased MMP. Treatment with lipoic acid does not have any effect on the ZnO NP–decreased MMP in the testes of rats.

Oxidative stress has been known to play a significant role in metal-induced toxic effects. The imbalance between the free radicals and antioxidants such as superoxide dismutase (SOD), catalase (CAT), and glutathione peroxidase (GPX) may lead to oxidative stress and finally organ toxicity (Ho et al., 2013; Ighodaro and Akinloye, 2018). Free radicals or reactive species through oxidative stress have been implicated in the incidence and progression of several health conditions such as atherosclerosis, diabetes, cancer, neurodegenerative disorders, cardiovascular disorders, and other chronic conditions (Sanders et al., 2009). We also observed an increase in oxidative stress in the nervous, immune, and male reproductive systems of lead- and ZnO NP–exposed group of animals. Treatment with alpha-lipoic acid reduced lead- and ZnO NPs–induced oxidative stress, suggesting its antioxidant potential.

In order to further understand the other mechanisms involved in the toxicity of lead and ZnO NPs, we measured the levels of caspase-3 which are involved in the apoptosis process. Caspase-3 is activated both in response to extrinsic and intrinsic cell death pathways. Our results demonstrated that in the lead-administered group, a significant increase in the levels of caspase-3 in both spleen and brain tissues was noted which signifies the cell death mechanism (Porter and Jänicke, 1999). However, the relative organ weight index showed no significant differences between the groups (Table 1). The metal concentration data suggested (Table 2) that the blood lead and zinc levels were increased significantly in lead and zinc exposed groups, while protection was noted in blood upon administration of α‐lipoic acid on lead levels, which decreases significantly, hence it is in sync with our blood ALAD data that shows a chelating property of α‐lipoic acid.

**TABLE 1:** Relative organ weight ratio and body weight in treated rats.

	Con	Pb	Pb ± LA	ZnO NPs	ZnO NPs ± LA	Pb ± ZnO NPs	Pb ± ZnO NPs ± LA
**Body weight (g)**	271 ± 10.8	290.6 ± 5.28	306 ± 18.0	286.1 ± 7.73	303 ± 12.9	288.6 ± 18.1	312.6 ± 8.7
**Spleen weight (g)**	1.015 ± 0.17	1.127 ± 0.09	0.995 ± 0.04	1.000 ± 0.04	0.821 ± 0.03	1.11 ± 0.13	1.097 ± 0.08
**Relative spleen weight (%)**	0.367 ± 0.03	0.388 ± 0.03	0.328 ± 0.02	0.349 ± 0.01	0.272 ± 0.01	0.384 ± 0.03	0.352 ± 0.03
**Brain weight (g)**	1.709 ± 0.14	1.846 ± 0.03	1.875 ± 0.038	1.848 ± 0.049	1.757 ± 0.058	1.782 ± 0.084	1.768 ± 0.072

Abbreviations: Wt, weight; g, gram. All the values are expressed as mean ± SEM (n = 4). There was no statistical significance among the groups. Relative organ weight = weight of the organ/body weight*100.

**TABLE 2:** Blood metal level (µg/ml blood) in rats. (A) Lead. (B) Zinc. All the values are expressed as mean ± SEM (n = 5). The statistical significance was considered at *p* < 0.05, and statistical analysis was performed using one-way ANOVA, followed by Tukey’s test. *p < 0.05, *p* < 0.05 vs. lead; ^c^p < 0.001 vs. ZnO NPs; ^b^p < 0.01 vs. Pb + ZnO NPs.

	Control	Pb	Pb + LA	ZnO NPs	ZnO NPs + LA	Pb + ZnO NPs	Pb + ZnO NPs + LA
A. Lead	0.09 ± 0.002	0.45 ± 0.02*	0.34 ± 0.002a	0.11 ± 0.002	0.09 ± 0.002	0.035 ± 0.0024	0.027 ± 0.0027
B. Zinc	0.8 ± 0.03	0.53 ± 0.03*	0.55 ± 0.0033	0.71 ± 0.003c	1.51 ± 0.003c	1.52 ± 0.0031c	1.49 ± 0.003c

Histological studies are useful for examining the microscopic anatomy of biological tissues. Following earlier reports, our investigation also showed that exposure to lead- and ZnO NP–induced histological changes in organs. The cognitive function can again be assessed by histopathological examination based on the facts that lead was already known to target the hippocampus and that lead inhibits NMDAR function. Histopathological examination revealed alteration in the hippocampus and the cortex structure. In Figure 15, the CA2 and CA3 regions showed disturbed cellular density in both lead-treated and ZnO nanoparticle–treated groups whereas α-lipoic acid–treated groups did not exhibit evident disturbed cellular density. The lead-exposed group of animals did not show alteration of biochemical and hematological parameters; however, histological alterations in the testis were observed. The histopathological damage caused by the administration of lead is already reported (Kumar and Devi, 2019). Talebi et al. (2013) have reported the cytotoxic effect of zinc oxide nanoparticles in testicular cells. The lipoic acid treatment has shown an attenuating effect on the lead- and zinc oxide nanoparticles–induced histological alterations. Rafiee et al. (2019) have reported the protective effect of lipoic acid in the reproductive organs of experimental animals.

The mechanisms involved in the protective effects against lead and zinc oxide nanoparticles can be postulated depending on the previous studies. ALA improves the redox state of the plasma and shows a protective effect on oxidative stress–induced apoptosis (Serhiyenko et al., 2018). One study of α-LA against hydrogen peroxide–induced toxicity in human lymphocytes showed it to be an ideal compound that has profound protective effects on oxidation, inflammation, and apoptosis (Rahimifard et al., 2015). Earlier studies on the beneficial role of alpha-lipoic acid against metals/metalloids such as arsenic, cadmium, mercury, and gold have shown the role of upregulated Nrf2 and GPx1 and downregulated Keap1 (Grunert, 1960; Saleh et al., 2017). The prophylactic role of α-lipoic acid against the toxic effect of zinc oxide nanoparticle (ZnO NP)–induced metabolic disorder, inflammation, and DNA damage in rat livers has been reported. Here, ALA ameliorated metabolic and immune disorders related to liver damage and modulated the previously measured parameters (Al-Rasheed et al., 2014). ALA was used as both antioxidant and chelating agent against lead-induced bone marrow toxicity because of its ability to scavenge free radicals and regenerate other antioxidants like SOD, GST, GPx, and CAT from their radical or inactive forms, and it has lead-chelating activity (Haleagrahara et al., 2011).

There has been a lot of cross talk between the peripheral and CNS immune components (Liu et al., 2020). The clearance system existed in the CNS prevents the accumulation of toxic peptides, and this is regulated by innate immune cells (Ordovas-Montanes et al., 2015). The interplay between central and peripheral immune systems works synergistically and involves inflammation and related pathologies (Su and Federoff, 2014). Thus, in our study, we reported the effect on both the nervous and immune systems. Further studies are required to prove the mechanisms involved in this cross talk.

## Conclusion

The present study reports the promising role of alpha-lipoic acid against lead-induced and ZnO NP–induced alerted immunological, neurological, and reproductive parameters in experimental rats. The therapeutic role of alpha-lipoic acid may be attributed to its multifaceted activities such as chelation, antioxidative, anti-inflammatory, and antiapoptotic activities. The exact molecular mechanism needs to be further investigated.

## Data Availability

The raw data supporting the conclusion of this article will be made available by the authors, without undue reservation.

## Abbreviations

AChE, Acetylcholinesterase; AIDS, Acquired immunodeficiency syndrome; ATP, Adenosine triphosphate; BSA, Bovine serum albumin; CNS, Central nervous system; DCFDA, 2′, 7’–dichlorofluorescin diacetate; DTNB, 5, 5′-dithiobis-(2-nitrobenzoic acid); ELISA, Enzyme-linked immunosorbent assay; GSH, Glutathione; LD50, Lethal dose, 50%; NADH, Nicotinamide adenine dinucleotide (NAD) + hydrogen (H); NBT, Nitro blue tetrazolium chloride; NMDA, N-methyl-d-aspartate; PMS, Phenazonium methosulphate; ROS, Reactive oxygen species; SOD, Superoxide dismutase; TBARS, Thiobarbituric acid reactive substances; ZnO NPs, Zinc oxide nanoparticles
